# Subcutaneous Construction of Engineered Adipose Tissue with Fat Lobule-Like Structure Using Injectable Poly-Benzyl-L-Glutamate Microspheres Loaded with Adipose-Derived Stem Cells

**DOI:** 10.1371/journal.pone.0135611

**Published:** 2015-08-14

**Authors:** Wentao Sun, Jianjun Fang, Qi Yong, Sufang Li, Qingping Xie, Jingbo Yin, Lei Cui

**Affiliations:** 1 Department of Plastic and Reconstructive Surgery, Shanghai 9th People’s Hospital, Shanghai Jiao Tong University School of Medicine, Shanghai, People's Republic of China; 2 Department of Polymer Materials, Shanghai University, Shanghai, People's Republic of China; 3 College of Chemistry and Chemical Engineering, Hunan University, Changsha, Hunan, People's Republic of China; 4 Department of Hand Surgery, Zhejiang Provincial People’s Hospital, Hangzhou, Zhejiang, People's Republic of China; Texas A&M University Baylor College of Dentistry, UNITED STATES

## Abstract

Porous microcarriers were fabricated from synthesized poly(γ-benzyl-L-glutamate) (PBLG) polymer to engineer adipose tissue with lobule-like structure via the injectable approach. The adipogenic differentiation of human adipose-derived stem cells (hASCs) seeded on porous PBLG microcarriers was determined by adipogenic gene expression and glycerol-3-phosphate dehydrogenase enzyme activity. In vitro adipogenic cultivation was performed for 7 days, and induced hASC/PBLG complex (Adi-ASC/PBLG group) was subcutaneously injected into nude mice. Injections of PBLG microcarriers alone (PBLG group) and non-induced hASC/PBLG complex (ASC/PBLG group) served as controls. Newly formed tissues were harvested after 4 and 8 weeks. Generation of subcutaneous adipose tissue with typical lobule-like structure separated by fibrous septa was observed upon injection of adipogenic-induced hASC/microsphere complex. Adipogenesis significantly increased in the Adi-ASC/PBLG group compared with the control groups. The angiogenesis in the engineered adipose tissue was comparable to that in normal tissue as determined by capillary density and luminal diameter. Cell tracking assay demonstrated that labeled hASCs remained detectable in the neo-generated tissues 8 weeks post-injection using green fluorescence protein-labeled hASCs. These results indicate that adipose tissue with typical lobule-like structure could be engineered using injectable porous PBLG microspheres loaded with adipogenic-induced hASCs.

## Introduction

Engineering adipose tissue is a promising alternative to plastic and reconstructive surgery for restoring body contours in patients who lost contour because of surgical resections, trauma, or congenital abnormalities [1, 2]. Seeding three-dimensional scaffolds with regenerative cell populations and creating tissue substitutes that can be used to generate predictable and stable adipose tissue is a major strategy for adipose tissue engineering [3].

Adipose-derived stem cells (ASCs) are a readily and ideal cell source for adipose tissue engineering among the candidate seed cells because of their sufficient availability, minimally invasive procurement, high proliferation and adipogenic differentiation potential [4]. The ASC yield after expansion is relatively high and averages approximately 2 × 10^6^ cells per ml of lipoaspirate tissue [2].

Degradable scaffold is another important element in cell-based tissue engineering. Injectable microcarriers are an appropriate and ideal choice for adipose tissue engineering due to their simple implantation, ability to fill irregular defects, and low scarring risk [5]. Polypeptides or poly(amino acid)s and their copolymers are versatile synthetic biomaterials with unique biological characteristics that have been increasingly used in recent studies on developing new bioactive biomaterials. Polypeptide-derived copolymers have drawn considerable attention as surgical sutures, drug delivery vehicles, and scaffolds in tissue engineering because of their adjustable biodegradability, low immunogenicity, good biocompatibility, and excellent mechanical properties [6–8]. Synthetic poly(γ-benzyl-L-glutamate) (PBLG), whose polymer backbone has a degradable amide bond, exhibits great fabrication potential in various copolymers, such as polypeptides, because of its excellent solubility in various chemical moieties and most organic solvents [8].

Human adipose tissue is mainly composed of fat lobules, which are minimal essential units [9]. A fat lobule consists of 10^2^ to 10^3^ adipocytes with the size of millimeters. Every lobule is anatomically separated by fibrous septa, where terminal microcirculation supplies nutrition to adipocytes [10]. In addition, the septa functionally support the lobules, provide resistance to the enlarging lobules, and offer scaffold to which blood vessels, nerves, lymphatics attach, as these structures traverse the compartment and anchor the panniculus and skin to the body [11]. Increasing evidence has shown a close correlation between angiogenesis and adipose tissue development, in which the adipokines and cytokines secreted by adipocytes influence vascular homeostasis [12]. Angiogenesis inhibitor treatment selectively ablates adipose tissue in obese mice [13], and fat cells cannot develop without vascularization [14]. Based on the previously mentioned reasons, we speculated that injecting porous PBLG microcarriers populated with adipocytes would generate engineered adipose tissue with lobule-like structure and a blood supply system.

This study evaluated the adipogenic differentiation of the ASCs seeded on PBLG microcarriers in vitro and engineered adipose tissue by injecting hASC-loaded PBLG microcarriers in vivo.

## Materials and Methods

### Harvest, propagation, and multipotent differentiation of human ASCs (hASCs)

Fresh human lipoaspirates were obtained from five healthy patients (with the average age of 29 years) who underwent abdominal liposuction at the Department of Plastic and Reconstructive Surgery of Shanghai 9th People’s Hospital. All protocols of human tissue handling were approved by the Research Ethical Committee of the Hospital, and patients provided informed consent for the use of their tissue in research. Processed lipoaspirate cells were isolated and cultured as previously described [15, 16]. The isolated hASCs were cultured in low glucose Dulbecco’s modified Eagle’s medium (LG-DMEM; HyClone, USA) supplemented with 10% fetal bovine serum (FBS; HyClone, USA) and 1% penicillin–streptomycin (HyClone, USA) (designated growth medium, GM). The cells were maintained at 37°C in 5% humidified CO_2_. The hASCs at passage 2 were collected for cell seeding.

Following previously established methods [15], the hASCs from passage 2 expansion were cultured in adipogenic, osteogenic, and chondrogenic media to evaluate the multipotent differentiation of the cultured hASCs. The cells were cultured in GM as a control group. Adipogenically differentiated cells were stained with Oil Red O (Sigma, USA) followed by microscopic observation to visualize the red-stained oil droplets. After 3 weeks, the onset of osteoblast formation was evaluated by assessing calcium accumulation using Alizarin Red (Sigma, USA). After 3 weeks, the chondrogenic pellets were fixed and embedded in paraffin blocks and analyzed by hematoxylin–eosin (H&E) and collagen II (Abcam, USA) staining.

### Preparation and characterization of porous PBLG microcarriers

PBLG with a molecular weight of 302100 synthesized at a feeding molar ratio of monomer and initiator [M]/[I] of 50/1 was used in this study. In brief, 2.0 g of 6.6 mM BLG NCA was dissolved with 60.0 ml of dry 1,4-dioxane in a flame-dried flask, and then 1.52 ml of 0.10 M dicyclohexylamine in 1,4-dioxane solution was added under vigorous stirring at 15°C for 3 days ([M]/[I] = 50/1). The mixture was precipitated into an excess of diethyl ether (2/1, v/v). The obtained product was washed twice with diethyl ether and dried under vacuum at room temperature for 24 h, affording 80% to 89% yield.

Water-in-oil emulsion was prepared by emulsifying 3 ml of aqueous solution comprising aqueous gelatin (6.5 wt%) and poly(vinyl alcohol) (PVA; 0.1 wt%) in a 20 ml of PBLG solution (dissolved in methylene chloride, 0.2 g, 1 wt%) using a homogenizer at 14,000 rpm for 1 min. The prepared emulsion was mechanically stirred with 100 ml of 0.1 wt% PVA solution at room temperature for 3 min to form a double emulsion (water-in-oil-in-water emulsion). The double emulsion was immediately immersed in a pre-cooling ice-cold PVA solution (1000 ml, 0.1 wt%) and gently stirred for 24 h to remove methylene chloride. The obtained microcarriers were then gently stirred in a warm water bath at 37°C for 5 h to remove residual gelatin. The microcarriers were sequentially filtered through 50 and 80 mesh screens, and the microcarriers with 200μm to 355 μm diameter were washed three times with distilled water and collected for further use as previously described [17].

A scanning electron microscope (SEM) (JXA-840; JEOL, Japan) was used to observe the surface morphology of the porous microcarriers. The pore diameter and porosity of the PBLG microcarriers were measured using a mercury porosimeter.

### Adipogenesis of hASCs within porous PBLG microcarriers

The PBLG microcarriers were sterilized before cell seeding by soaking in 75% ethanol solution for 30 min and washing three times with phosphate buffered saline (PBS). Cell suspensions containing 5 × 10^6^ hASCs were mixed with 40 mg of PBLG microcarriers (about 1000 PBLG vehicles) in 10 ml of growth medium in each T-25 flask (Corning, NY, USA) (the seeded hASCs number that corresponds to microcarriers in the in vitro experiments was the same to that in the following in vivo experiments). The flasks were shaken at 75 rpm for 12 h in an incubator to evenly seed the cells on microcarriers, and 20 ml of growth medium was added for long-term culture.

The viability of the PBLG-attached hASCs was evaluated 48 h after seeding using a Live/Dead Double Stain Kit (Calbiochem, USA) according to the manufacturer’s instructions. The hASC-seeded microcarriers were fixed in 4% paraformaldehyde, stained with Hoechst 33258 (Sigma, USA) dye solution, and observed under confocal laser scanning microscope (LSM710; Carl Zeiss, Germany) to visualize the cellularity inside the microcarriers.

The cells were seeded for 7 days (designated day 0), and adipogenic differentiation was induced by replacing the cells in adipogenic differentiation medium (AM) containing LG-DMEM supplemented with 10% FBS, 0.5 mM 3-isobutyl-1-methylxanthine (IBMX; Sigma, USA), 200 μM indomethacin (Sigma, USA), 10 μM insulin (Sigma, USA), and 1 μM dexamethasone (Sigma, USA) [2]. The hASC/PBLG constructs were examined for cell proliferation, gene expression, and glycerol-3-phosphate dehydrogenase (GPDH) activity at indicated time points. The hASCs growing within the PBLG microspheres cultured in AM or GM were quantified by DNA assay using Hoechst 33258 dye as previously reported [18].

### Quantitative Real-time PCR

Total RNA was extracted from the samples using TRIzol (Invitrogen, USA) reagent according to the manufacturer’s instructions. RNA concentration was determined by measuring the optical absorbance of the samples at 260 nm. The extracted RNA sample (2 μg) was initially reverse transcribed for first strand cDNA synthesis using a PrimeScript 1st Strand cDNA synthesis kit (TaKaRa, China). The reactions were performed in a T3 thermocycler (Biometra, Germany). Real-time PCR was performed using a quantitative real-time amplification system (ABI PRISM 7500 Fast Real-time PCR System; Applied Biosystems, Foster City, CA, USA). SYBR Premix Ex Taq (Tli RNaseH Plus) (Takara, China) was used in each reaction. Adipogenic differentiation markers, namely, adipocyte Protein 2 (ap2), CCAAT/enhancer-binding protein alpha (C/EBP α), lipoprotein lipase (LPL), and peroxisome-proliferating activated receptor γ (PPAR γ), were evaluated (see Table 1). The relative expression level of each gene of interest was calculated by normalizing the quantified cDNA transcript level (cycle threshold) to that of GAPDH using the ABI PRISM 7500 Fast Real-Time PCR System.

**Table 1:** Primers for Real-Time Polymerase Chain Reaction

RNA	Primer	Sequences	Fragment size(bp)
**aP2**	Forward	GGCCAGGAATTTGACGAAG (19)	207
	Reverse	TCCCTTGGCTTATGCTCTCT (20)	
**C/EBP α**	Forward	CGGACTTGGTGCGTCTAAG(19)	147
	Reverse	CATTGGAGCGGTGAGTTTG(19)	
**LPL**	Forward	AAGCTGCCCACTTCTAGCTG(20)	249
	Reverse	ATCTCTTCTTTGGTCGGCGG(20)	
**PPAR γ**	Forward	TCTCTCCGTAATGGAAGACC (20)	474
	Reverse	GCATTATGAGACATCCCCAC (20)	
**GAPDH**	Forward	TGTTGCCATCAATGACCCCTT(21)	206
	Reverse	CTCCACGACGTACTCAGC(18)	

ap2: adipocyte Protein 2; C/EBP α: CCAAT/enhancer-binding protein alpha; LPL: lipoprotein lipase; PPAR γ: peroxisome-proliferating activated receptor γ; GAPDH: glyceraldehyde-3-phosphate dehydrogenase

### Subcutaneous injection of hASC/microcarrier complex

A total of 30 female nude mice, aged 6 weeks to 8 weeks and weighing 20 g to 25 g, were purchased from SLAC National Rodent Laboratory Animal Resources (Shanghai, China). The institutional review committee of Shanghai Jiao Tong University School of Medicine approved all animal study protocols. The mice were maintained under specific pathogen-free conditions and randomized into three groups of 10 mice each, namely, Adi-ASC/PBLG group (injected with adipogenic-induced hASC/PBLG complex), ASC/PBLG group (injected with non-induced hASC/PBLG complex), and PBLG group (injected with only PBLG microcarriers). Each injection consisted of 80 mg of PBLG microcarriers.

The complex was washed three times with sterile LG-DMEM medium (without FBS) and added with adequate LG-DMEM to make the final volume 0.5 ml before injection. The constructs were subcutaneously injected into the scalp of the nude mice using 18 gauge needles, while the animals were manually restrained.

After 4 and 8 weeks, 5 mice in each group were humanely killed, and neo-generated tissues were carefully dissected from the surrounding tissues and weighed. Tissue volume was measured using the liquid overflow method [19].

### Histological observation

The harvested specimens were fixed in 10% phosphate-buffered formalin, embedded in paraffin, and sectioned at 5 μm thickness for both H&E and Masson’s trichrome staining. Some harvested tissues were frozen in Tissue-Tek OCT freezing medium (Sakura Finetek Inc., Torrance, CA, USA) and sectioned at 8 μm thickness for Oil Red O staining. Blood vessel density and luminal diameter were measured according to a published method [20].

### Green fluorescence protein (GFP) labeling of hASCs

The subconfluent hASCs at passage 2 were transfected with GFP lentivirus vectors at 100 PFU/cell MOIs overnight. When the percentage of positive transfection exceeded 90%, the cells were seeded on PBLG microspheres and subcutaneously injected in nude mice for the cell tracking assay.

After 4 weeks and 8 weeks, newly formed tissues were harvested and flash-frozen in Tissue-Tek OCT freezing medium. The frozen sections were washed with PBS, stained with 5 μg/ml Hoechst 33258 dye solution, and observed using confocal laser scanning microscope.

### GPDH activity and hydroxyproline determination

GPDH activity was measured using a GPDH Kit (Clontech, MK426, USA) according to the manufacturer’s instructions. In brief, each sample was homogenized in 1 ml of 0.25 M sucrose solution at 4°C and disrupted with three 5-s sonication bursts, with intervals of cooling on ice. The sample was centrifuged at 16,000 ×*g* and 4°C for 10 min. The supernatant was transferred into a new 1.5 ml tube and centrifuged at 16,000 ×*g* and 4°C for an additional hour to isolate the cytosolic protein fraction, which includes GPDH. The supernatant was immediately assayed for GPDH activity according to the manufacturer’s instructions. Each sample was assayed in triplicate for total cytosolic protein by the Bradford method with an albumin standard to normalize the GPDH activity levels. The results are expressed as mU/mg protein (1 U = 1 μmol NADH/min).

The hydroxyproline content in the specimens was determined by an alkaline hydrolysis-based method using a hydroxyproline detection kit (Nanjing Jianchen Bioengineering Institute, Nanjing, China) according to the manufacturer’s instructions [21].

### Statistical analysis

All numerical data are expressed as mean ± standard deviation (SD). The data were analyzed by two-tailed Student's *t*-test for means analysis to compare two data groups or ANOVA to compare three or more data groups. A *P* < 0.05 was considered statistically significant (n = 3).

## Results

### Multipotent differentiation of hASCs

**Fig 1:**
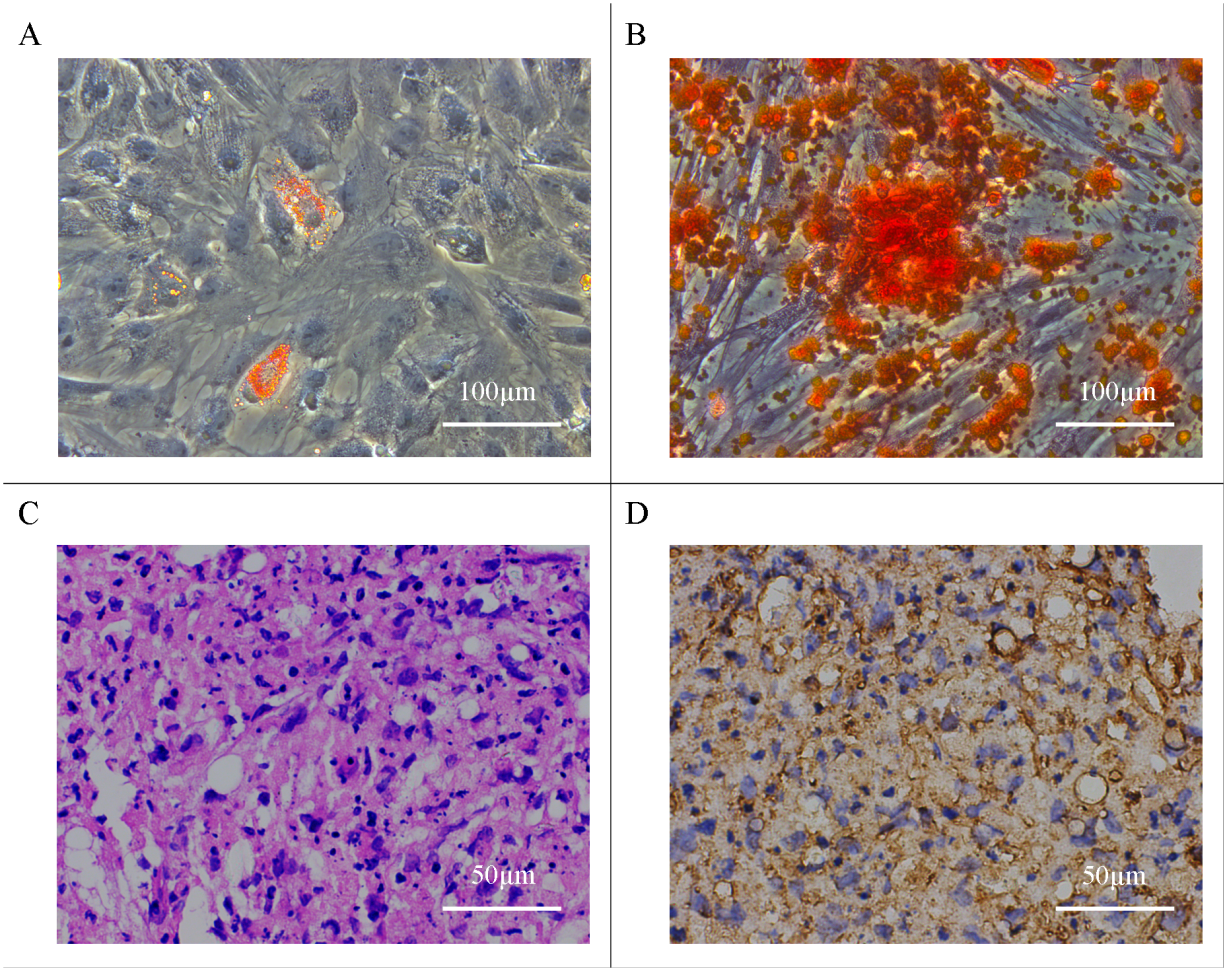
Multipotent differentiation of hASCs in vitro. (A) Oil Red O staining detected red-colored oil droplets in adipogenic differentiation of hASCs; (B) Alizarin Red detected calcium mineralization in osteogenic differentiation of hASCs; (C and D) For chondrogenic differentiation, histological and H&E staining results showed that cartilage lacunae were formed and expressed chondrocyte gene marker, collagen II.

In the present study, hASCs could be induced to differentiate along the adipogenic, osteogenic, and chondrogenic lineages using special cell culture media. Adipogenic differentiation was confirmed following the standard protocol and analyzed by Oil Red O staining. Red-colored oil droplets in adipogenic cultures were observed (Fig 1A). Cells with bone-forming capacity were examined by Alizarin Red (Fig 1B). For chondrogenic differentiation, histological and H&E staining results showed that cartilage lacunae were formed (Fig 1C) and expressed the chondrocyte gene marker, collagen II (Fig 1D).

### Attachment, migration, and proliferation of hASCs within PBLG microspheres

**Fig 2:**
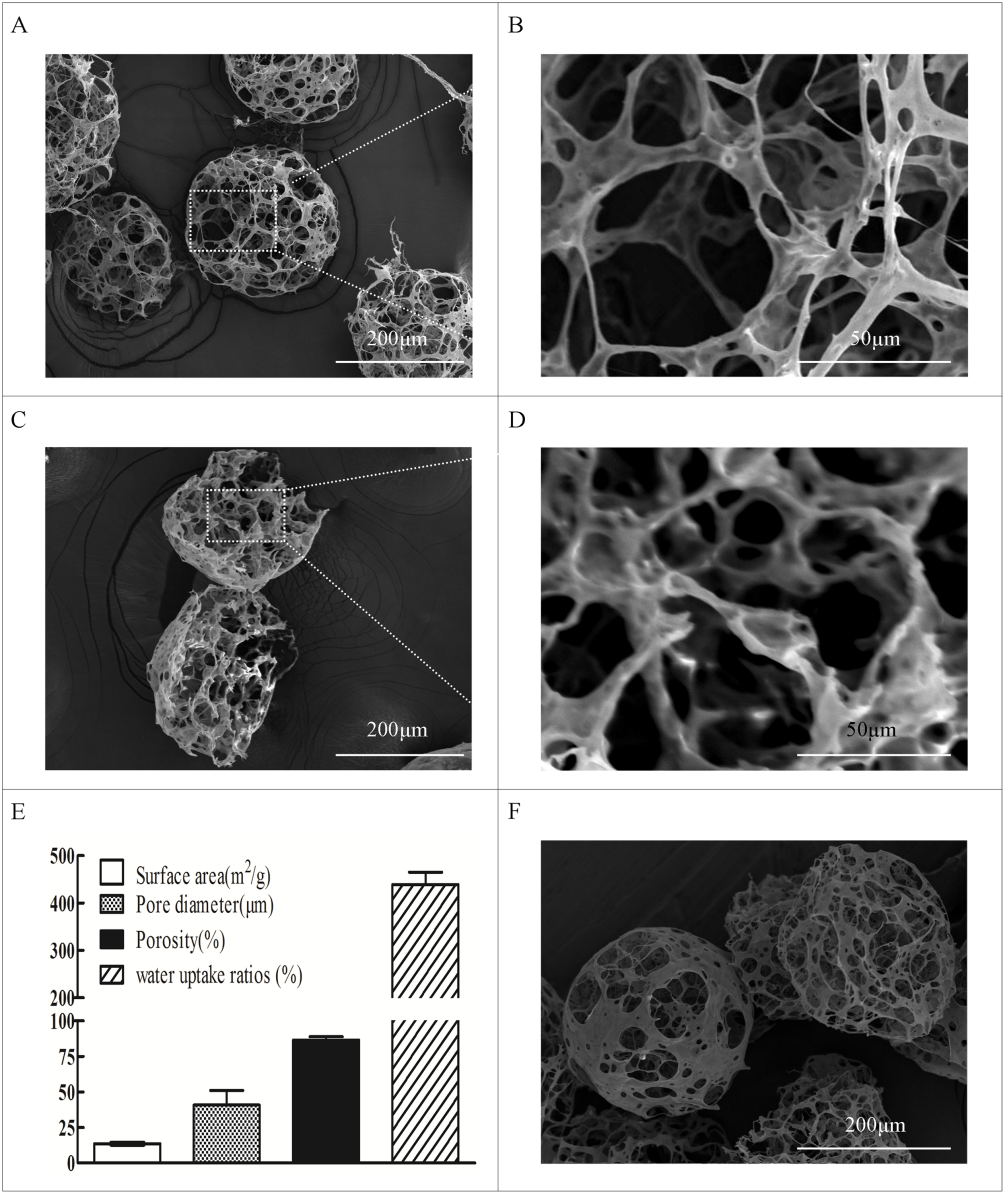
Physical characteristics of PBLG microspheres. (A, B) SEM examination of the whole porous PBLG microcarriers. (C, D) Cross-sectional view of porous PBLG microcarriers. (E) Porosity and pore diameters of PBLG microcarriers (n = 3). (F) SEM examination of injected porous PBLG microcarriers.

The microcarriers exhibited homogenous spherical morphology with 238.5 ± 21.9 μm diameter (Fig 2A). The SEM observation showed that the highly porous structure was interconnected with 40.3 ± 9.8 μm pore size and 84.2 ± 1.39% porosity (Fig 2B–2E, S1 Table). After injection through an 18-gauge needle, the microcarriers maintained their spherical morphology, and interconnected pore structure (Fig 2F), which indicated that porous PBLG microspheres could withstand forces on injection.

**Fig 3:**
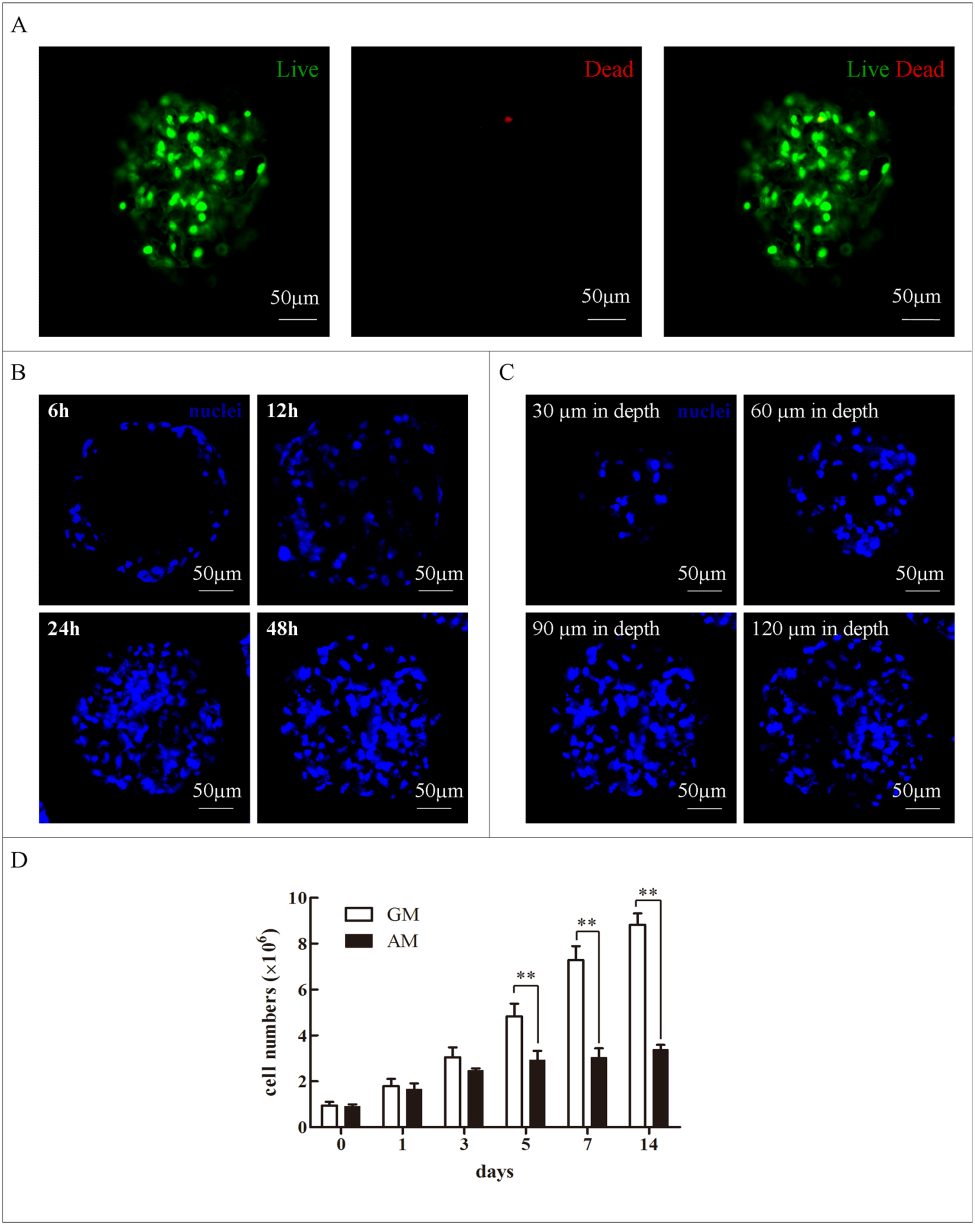
Biological characteristics of hASCs growing within porous PBLG microspheres. (A) Confocal images of the live (green)/dead (red) assay for the hASCs growing in microcarriers 48 h post-seeding. (B) Confocal laser microscopy observation of Hoechst33258-stained hASCs growing within the porous PBLG microcarriers at 6, 12, 24, and 48 h. (C) Confocal laser microscopy observation of Hoechst33258-stained hASCs at the indicated depth in the microsphere after cell seeding for 48 h. (D) hASC proliferation within PBLG microspheres maintained in adipogenic medium (AM) or growth medium (GM) for 14 d (n = 3). *P < 0.05; **P < 0.01.

The fluorescence images of the live (green)/dead (red) assay indicated that most cells in the porous constructs were viable after 48 h of seeding (Fig 3A). The hASC-attached microcarriers were stained with Hoechst 33258 dye solution at 6, 12, 24, and 48 h post-seeding to investigate the progressive spatial hASC distribution in the microspheres. Fig 3B shows that most cells were located on the microcarrier surface after seeding, and hASCs eventually spread throughout the microcarriers by 48 h. The hASC-attached microspheres were observed by confocal microscopy at depths of 30, 60, 90, and 120 μm at 48 h post-seeding to determine whether the seeded hASCs infiltrated the microsphere region. Fig 3C shows that the inoculated hASCs infiltrated into the central region of each microsphere layer. The average diameter of the PBLG microspheres used in this study was 238.5 ± 21.9 μm. Thus, the seeded cells populated the whole PBLG microsphere regions 48 h post-seeding.

The cells were counted at 0, 1, 3, 5, 7, and 14 days after seeding to assess hASC proliferation growing within the microcarriers. The number of the hASCs cultured in either GM or AM continuously increased for 14 d. However, the hASCs cultured in AM exhibited a relatively lower proliferation potential than those in GM (Fig 3D, S2 Table).

### Adipogenic differentiation of the hASCs within microspheres

**Fig 4:**
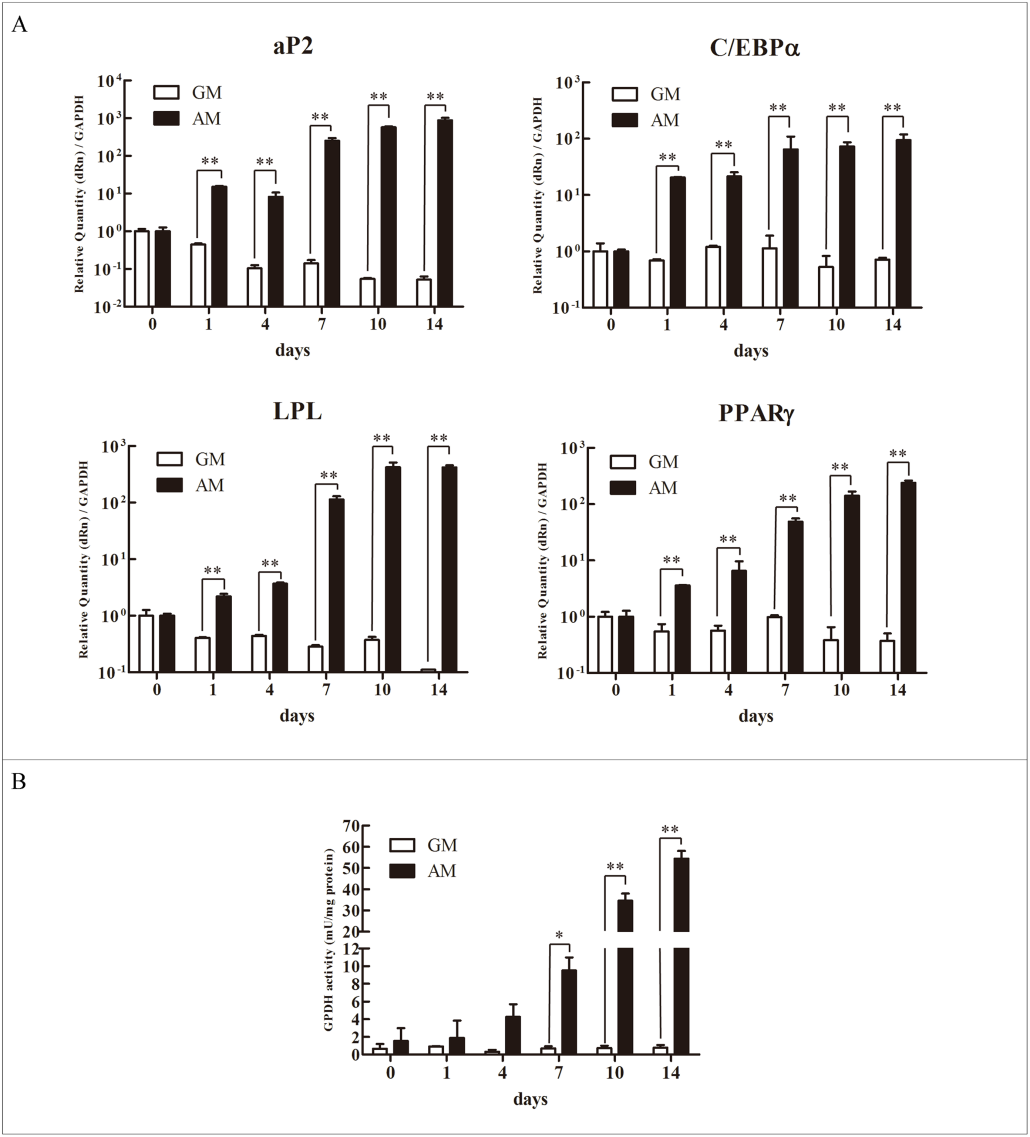
Adipogenic differentiation of hASCs within PBLG microspheres. (A) Real-time PCR detection of the expression of adipogenic genes, including aP2, C/EBP α, LPL, and PPAR γ, at the indicated time points (n = 3). (B) GPDH enzyme activity of the hASCs growing within the PBLG microspheres maintained in adipogenic medium (AM) or growth medium (GM) (n = 3). *P < 0.05; **P < 0.01.

Real-time PCR analysis revealed that the expression levels of adipogenic gene, including aP2, C/EBP α, LPL, and PPAR γ, in the adipo-induced ASC/PBLG complex constantly increased, which were significantly higher than those in the non-induced ASC/PBLG complex (Fig 4A, S3, S4, S5 and S6 Tables).

The GPDH activity of the Adi-ASC/PBLG group began increasing at 4 days and reaching 54.45 ± 3.62 mU/mg by day 14, which is nearly 72-fold higher than that in the ASC/PBLG group (0.76 ± 0.33 mU/mg) (Fig 4B, S7 Table).

### Characterization of the newly formed tissues in vivo

#### Macroscopic examination

**Fig 5:**
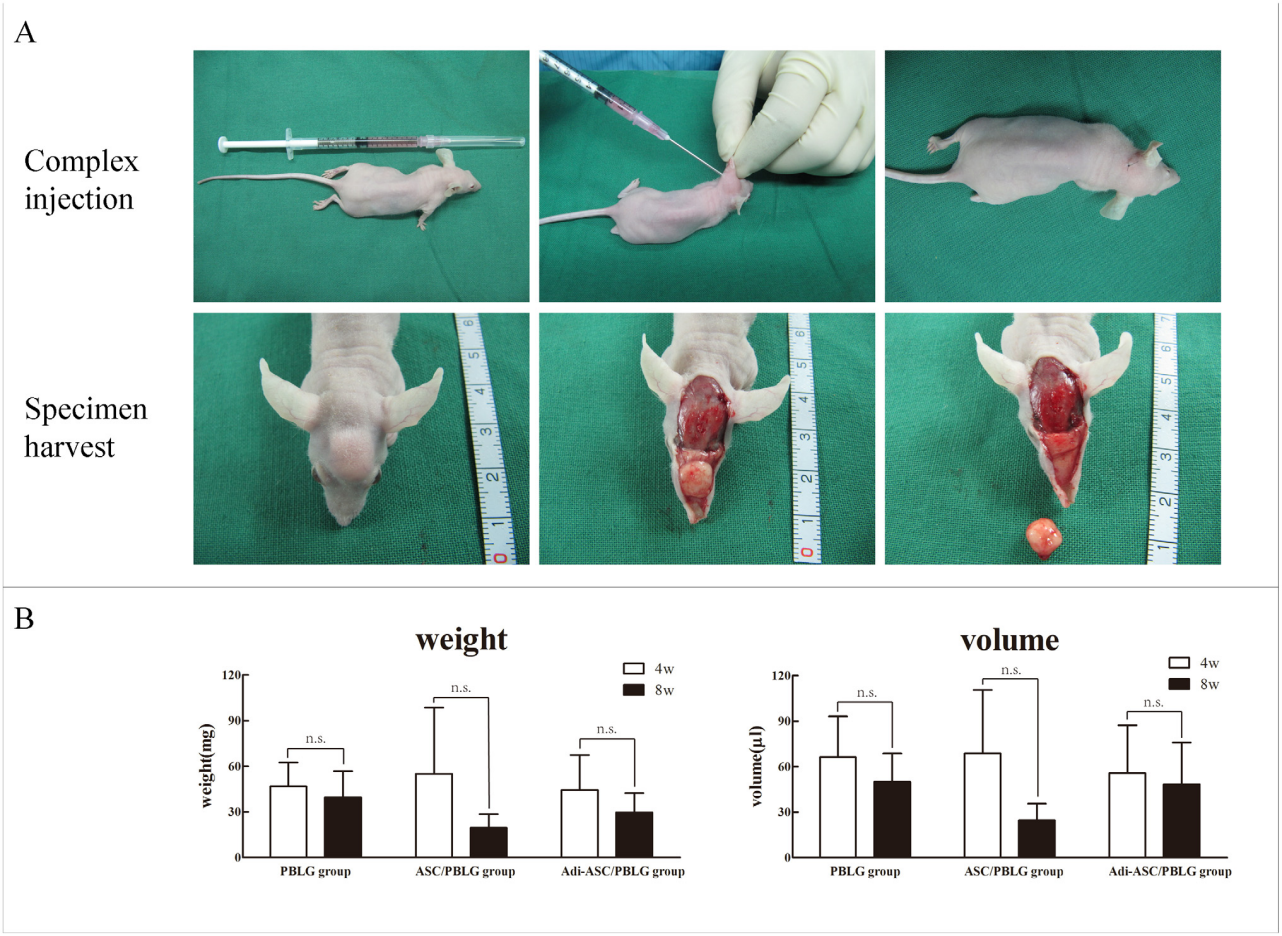
Construction, harvest and analysis of the neo-generated tissues. (A) Subcutaneous injection of hASC/PBLG microsphere complex and harvest of neo-generated tissue after 8 weeks. (B) Weight and volume analysis of the neo-generated tissues (n = 3) (*P* > 0.05). n.s. = no statistical significance.

To determine the in vivo efficacy of PBLG microcarriers as a delivery system for adipose tissue engineering, we subcutaneously injected adipogenic-induced hASC/PBLG complex (Adi-ASC/PBLG group) under the scalp of the nude mice. Microcarriers alone (PBLG group) and non-induced hASC/PBLG complex (ASC/PBLG group) served as the controls. A well-defined subcutaneous lump was observed in all three groups at 4 and 8 weeks post-injection (Fig 5A). The tissue harvested from the Adi-ASC/PBLG group was light yellow and semispherical at either 4 or 8 weeks post-injection. The average weight and volume of the neo-generated tissue among each group were not statistically significant at either 4 weeks or 8 weeks after implantation (Fig 5B, S8 and S9 Tables).

#### Histological observation

**Fig 6:**
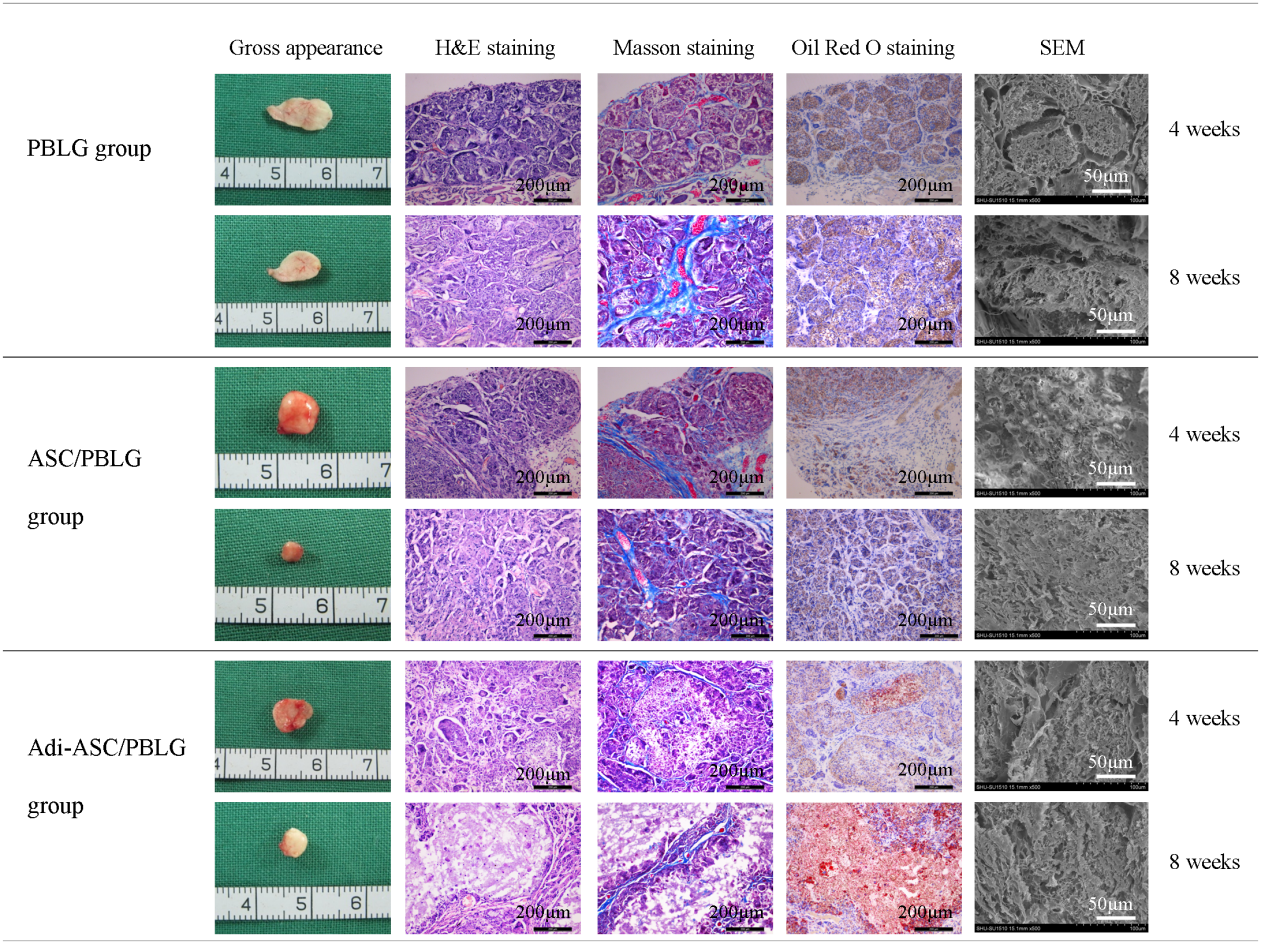
H&E, Masson’s trichrome, Oil Red O staining, and scanning electron microscope (SEM) examination of the neo-generated tissue in the three groups at 4 and 8 weeks post-injection. (A) PBLG group: injection of PBLG microsphere alone; (B) ASC/PBLG group: injection of non-induced hASC/PBLG complex; and (C) Adi-ASC/PBLG group (4 weeks): injection of adipogenic-induced hASC/PBLG microsphere complex.

The neo-generated tissue was histologically composed of many lobule-like structures separated from each other by fiber tissue. Masson's trichrome staining revealed that the collagen fibers deposited in the septa with in-growth of blood vessels contained closely packed erythrocytes. Meanwhile, most pores in the microspheres were occupied by infiltrating fibrous tissue. The boundary of the ASC-seeded microspheres could no longer be clearly detected at 8 weeks. The progressive development of adipose tissue in the Adi-ASC/PBLG group upon injection was further characterized by Oil Red O staining, showing intracellular lipid accumulation. However, adipose could not be observed in the PBLG and ASC/PBLG groups (Fig 6). Further calculation showed that the size of the newly formed fat lobule was 1.1 ± 0.5 mm. Through SEM examination, the boundaries of the implanted microspheres were only recognizable in the PBLG group but not in other two groups at 4 weeks or 8 weeks post-injection, which might be due to the growth of cells and the deposition of extracellular matrix masking the microspheres.

**Fig 7:**
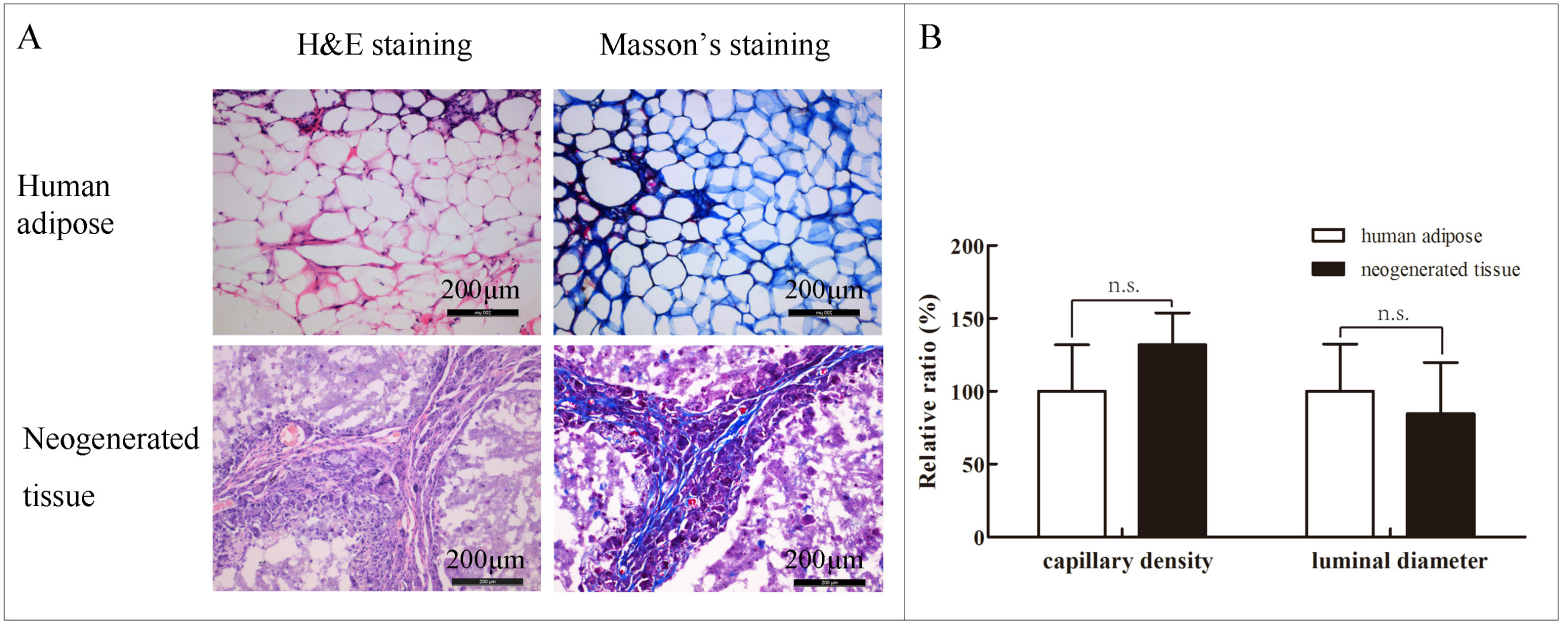
Histological analysis of normal human adipose and neo-generated tissues harvested from the Adi-ASC/PBLG group 8 weeks post-injection. (A) H&E and Masson’s trichrome staining of normal human adipose and neo-generated fat tissues; (B) Comparative analysis of the number of vessel lumina and luminal diameters between normal human adipose and neo-generated tissues (n = 3) (*P* > 0.05). n.s. = no statistical significance.

To provide more insights on the vascularization of the neo-generated adipose tissue, we determined the capillary density and luminal diameter of the samples harvested from the Adi-ASC/PBLG group at 8 weeks post-injection. Fig 7B shows that the average capillary density and luminal diameter in the engineered fat were 35.9 ± 5.9 lumens/mm^2^ and 21.4 ± 8.9 μm, respectively (S10 Table). These results indicate no significant difference from those in normal fat, which were 27.2 ± 8.7 lumens/mm^2^ and 25.3 ± 8.2 μm, respectively.

**Fig 8:**
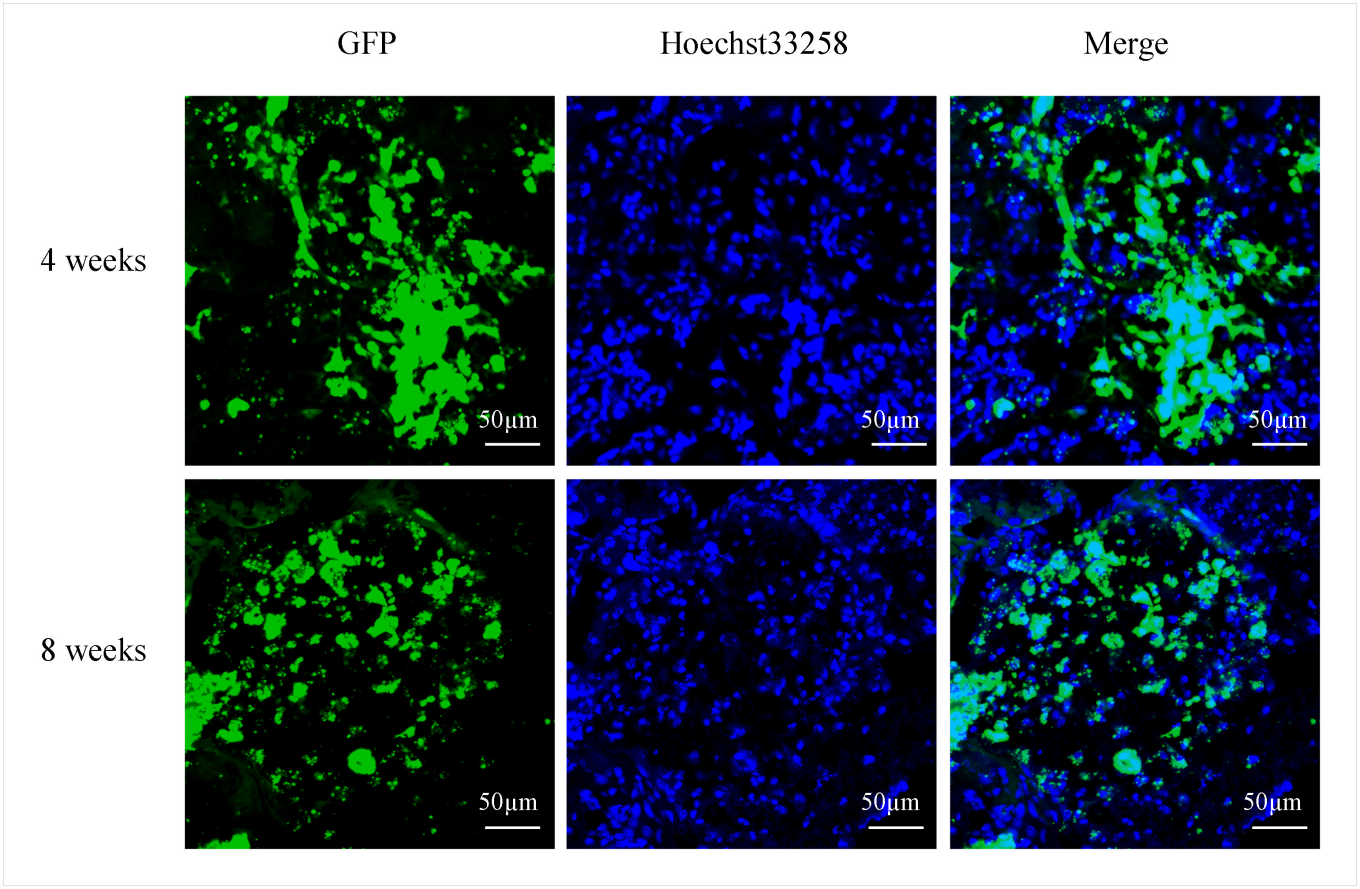
GFP-labeled hASC detection in the engineered adipose tissues by confocal laser microscopy observation at 4 and 8 weeks post-injection. Cell nuclei were counterstained with Hoechst 33258 dye.

The cells were labeled with fluorescent GFP and traced at 4 and 8 weeks post-injection to determine whether the neo-generated adipose tissue was derived from the implanted hASCs. Most of the spheres were occupied with GFP-labeled cells. The septa exhibited little GFP-labeled cells, indicating that the formed fibrous septa and the capillaries within the generated tissue were derived from the surrounding host tissue (Fig 8).

#### Biochemical analysis

**Fig 9:**
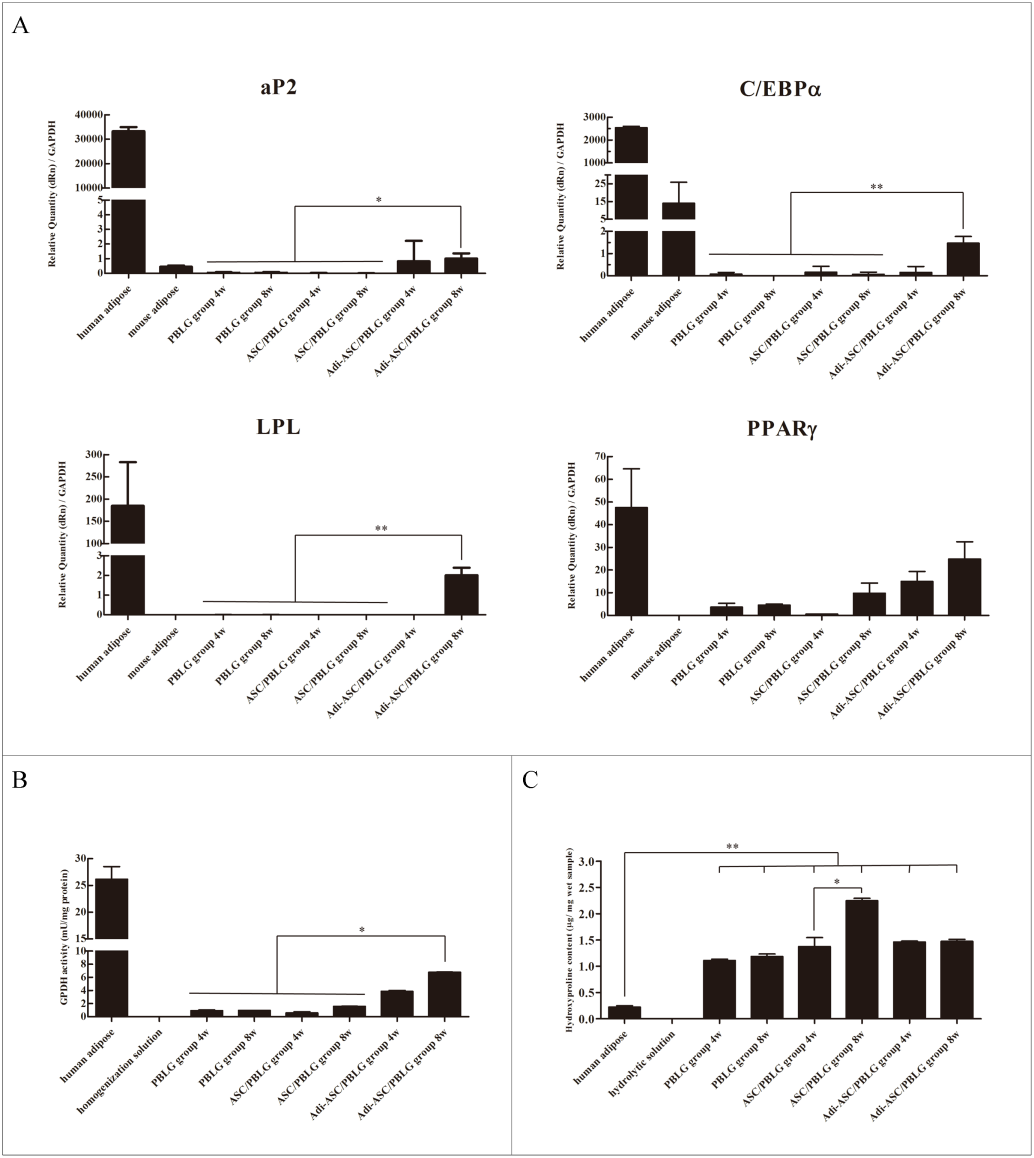
Biochemical analysis of neo-generated tissue from different groups after 8 weeks of treatment. (A) qRT-PCR analysis of adipogenic gene expression from the harvested tissue (n = 3). (B) GPDH enzyme activity of the neo-generated tissues harvested from the three groups at 4 and 8 weeks post-injection (n = 3). (C) Hydroxyproline content in the neo-generated tissue. Human adipose was used as the positive control (n = 3). *P < 0.05; **P < 0.01.

Real-time PCR was performed to analyze the expression of master adipogenic genes, C/EBP α and PPAR γ, and later markers, ap2 and LPL, in the newly formed tissues at 4 and 8 weeks post-injection. The expression of these adipogenic genes was significantly higher in the Adi-ASC/PBLG group than that in the PBLG and ASC/PBLG groups (Fig 9A, S11 Table).

The quantitative measurement of in vivo GPDH enzyme activity demonstrated the formation of engineered adipose tissue. Fig 9B shows that the GPDH activities in the Adi-ASC/PBLG group were 3.86 ± 0.14 and 6.77 ± 0.02 mU/mg at 4 and 8 weeks, respectively (S11 Table). By contrast, GPDH activities were maintained at relatively lower levels in both the PBLG and ASC/PBLG groups, both of which are not statistically different.

The collagen content within the newly formed tissues was investigated because collagen network greatly contributes to the structural maintenance of adipose tissue. Fig 9C shows that the hydroxyproline content in the ASC/PBLG group was highest among all the three groups at 8 weeks post-injection (S11 Table). However, the collagen content in normal human adipose tissue was significantly lower than that in the three experimental groups, indicating in-growth of fibrous tissue from the surrounding tissue.

## Discussion

This study demonstrates that the novel porous PBLG microspheres possess good biocompatibility for proliferation of seeded hASCs. Living engineered adipose tissue with typical lobule-like structure was successfully generated by injecting PBLG microspheres loaded with ASCs.

Many natural biomaterials, such as gelatin, and synthetic polymers, such as polylactic acid, have been evaluated as cellular delivery systems in adipose tissue engineering. Compared to their natural counterparts, synthetic polymers can be tailored to meet in vivo mechanical and chemical requirements [22]. Synthetic PBLG, as a degradable polypeptide, is widely used in biomedical applications due to its adjustable biodegradability, low immunogenicity and good biocompatibility [6–8]. PBLG and its derivatives have been synthesized as implantable tissue scaffolds for bone tissue engineering [8, 23, 24]. Qian et al. [8] developed bimodal porous PBLG polypeptide scaffolds using a combination of in situ ring-opening γ-benzyl-L-gIutamate N-carboxyanhydride polymerization and a biotemplating method. The result showed improved osteogenic differentiation of the preosteoblast cell line seeded in the scaffold. To our knowledge, only a few studies about using injectable PBLG microspheres as delivery vehicles in tissue engineering have been performed [17].

Given that the limit of the oxygen diffusion capacity in dense cellular structures is typically 150 μm to 250 μm [25], we fabricated a homogenous spherical microcarrier with an average diameter of 238.5 ± 21.9 μm. The fabricated porous microcarriers exhibited a highly porous structure that was favorably interconnected with an average pore size of 40.3 ± 9.8 μm. Huang et al. [26] demonstrated that high interconnectivity and large pore size in poly(D, L-lactic-co-glycolic acid) beads could facilitate the distribution, in-growth, and differentiation of the human amniotic fluid stem cells seeded throughout the porous microsphere. The seeded ASCs in the present study were distributed in the innermost region of PBLG microspheres as early as 24 h, consistent with the study by Huang et al [26].

Adipose tissue engineering is a promising alternative to generate functional fat tissue substitutes [27]. It requires biomaterials with suitable mechanical properties and degradability rates. Brandl et al. [27] evaluated the effects of hydrogels degradability on tissue development and demonstrated that enzymatically degradable hydrogels promote the formation of coherent adipose tissue-like structures featuring more mature unilocular adipocytes than non-degradable hydrogels. The degradation rate is necessary to be adapted to the tissue formation [28]. Choi et al. [4] studied the combination of injectable PLGA spheres and hASCs in constructing adipose tissue and demonstrated that ASCs attached to PLGA could fully differentiate into mature adipocytes 8 weeks after injection. The degradation of PBLG microspheres and the adipogenesis of seeded hASCs in vivo were observed in the present study, showing that the adipose tissue generated by engrafted ASCs occupied concurrently the space filled by biomaterials with PBLG microsphere degradation. The degradation of PBLG microspheres in vitro and in vivo were evaluated in our previous study [17]. However, it was still unknown when the PBLG microspheres in vivo were degraded completely. And next step prepared to do is to clarify that question.

During adipogenesis progression, several adipogenesis-related genes involved in adipocyte differentiation have been identified. Among these genes, PPARγ and C/EBPα are two main transcription factors that are critical for adipogenesis. These factors act cooperatively in adipogenic differentiation by activating the expression of one another, regulating the expression of other adipocyte-specific genes critical to adipogenesis, lipid metabolism, and lipid uptake, and therefore inducing fat cell differentiation [3, 29]. The LPL secreted by mature adipocytes controls lipid accumulation, catalyzes triacylglycerol hydrolysis, and is abundant in adipose tissue. LPL is often considered as an early biochemical marker of adipocyte differentiation [3]. aP2 is a key regulator of intracellular transport and metabolism of fatty acids and a predominant fatty acid-binding protein in adipose tissue [3]. The expression of aP2 is almost exclusively confined to fat tissue and adipogenic cell lines, and highly regulated during adipocyte differentiation [30]. GPDH is a cytoplasmic enzyme involved in the triglyceride biosynthesis pathway, coverting dihydroxyacetone phosphate into glycerol 3-phosphate [31]. GPDH activity increases during fat cell maturation and terminal adipogenic differentiation [32]. Thus, the upregulation of these markers and GPDH activity in engineered adipose tissue is a clear indication of a physiologically relevant progression toward adipogenesis.

Fat lobule, which is separated by interlobular fibrous septa, is the minimal essential unit of adipose tissues. The presence of fat lobules interspersed between fibrous and glandular components help determine the contour, bulk, and softness of the soft breast [9]. The ECM in adipose tissue protects cells, acts as depots for accumulation of hormones and growth factors, and provides mechanical tensile strength to soft tissue [33]. The hydroxyproline content in neo-generated adipose tissue at 8 weeks post-injection was significantly higher than that in native adipose tissue, indicating in-growth of fibrous tissue from the surrounding tissue. The capillary density and luminal diameter of blood vessels in the neo-generated tissue were determined because the in vivo engraftment of engineered fat is mostly dependent on in-growth of host-derived blood vessels. The angiogenesis in the neo-generated tissues was comparable to that in normal ones, indicating sufficient blood supply for engineered fat survival. Therefore, although the lobule size of the newly formed tissue is smaller than that of the native human fat lobules, the neo-generated tissue mimics the structure and ECM deposition of native fat lobules [34].

## Conclusions

The porous PBLG microspheres prepared in this study provide suitable structure and biocompatibility for seeded hASCs proliferation and adipogenic differentiation. Subcutaneous fat tissue with typical lobule-like structure was successfully generated in nude mice by injecting microspheres loaded with adipogenic hASCs. The injectable PBLG microspheres allow easy manipulation and minimally invasive surgical procedures in adipose tissue engineering, focusing on the potential clinical application of this approach for soft tissue augmentation and contour improvement.

## Supporting Information

S1 Table Physical parameters of PBLG microcarriers. (TIF)

S2 Table hASC proliferation within PBLG microspheres maintained in adipogenic medium (AM) or growth medium (GM) for 14 d. (TIF)

S3 Table Real-time PCR detection of the expression of aP2 at the indicated time points. (TIF)

S4 Table Real-time PCR detection of the expression of C/EBP α at the indicated time points. (TIF)

S5 Table Real-time PCR detection of the expression of LPL at the indicated time points. (TIF)

S6 Table Real-time PCR detection of the expression of PPAR γ at the indicated time points. (TIF)

S7 Table GPDH enzyme activity of the hASCs growing within the PBLG microspheres maintained in adipogenic medium (AM) or growth medium (GM). (TIF)

S8 Table Weight analysis of the neo-generated tissues. (TIF)

S9 Table Volume analysis of the neo-generated tissues. (TIF)

S10 Table Comparative analysis of the number of vessel lumina and luminal diameters between normal human adipose and neo-generated tissues (TIF)

S11 Table Biochemical analysis of neo-generated tissue from different groups after 8 weeks of treatment. (TIF)
