# Supplementary figures and images for: Subcutaneous Construction of Engineered Adipose Tissue with Fat Lobule-Like Structure Using Injectable Poly-Benzyl-L-Glutamate Microspheres Loaded with Adipose-Derived Stem Cells
Source: PLoS One. 2015 Aug 14;10(8):e0135611. doi: 10.1371/journal.pone.0135611 (PMC4537260; doi:10.1371/journal.pone.0135611)

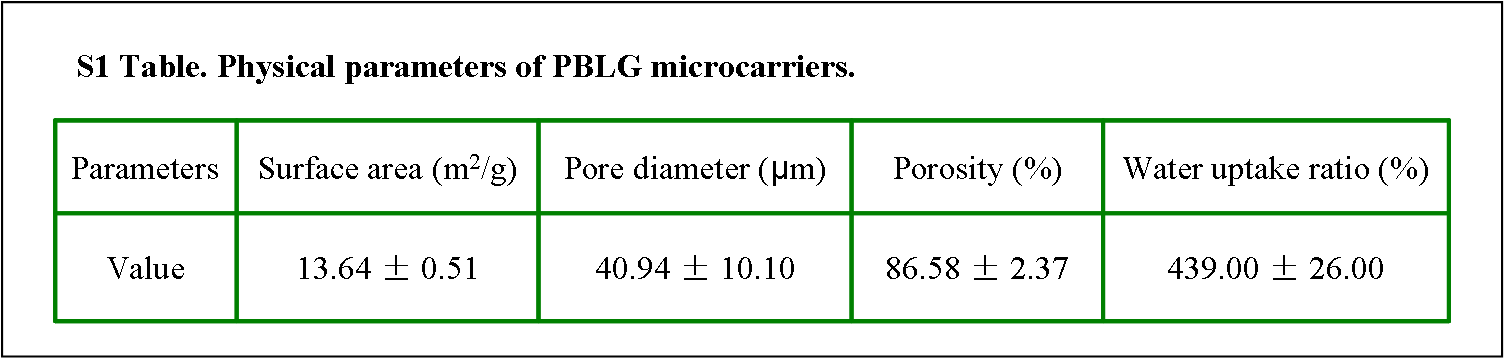

Supplement: S1 Table — (TIF) [file pone.0135611.s001.tif]

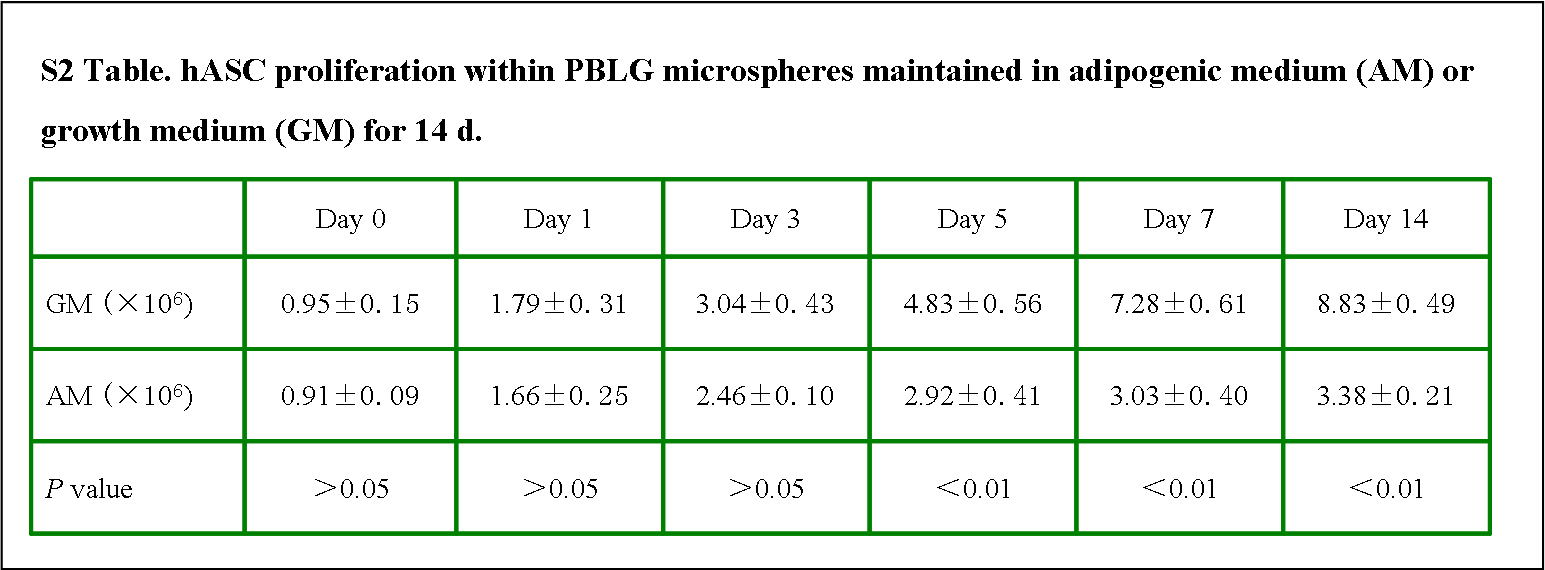

Supplement: S2 Table — (TIF) [file pone.0135611.s002.tif]

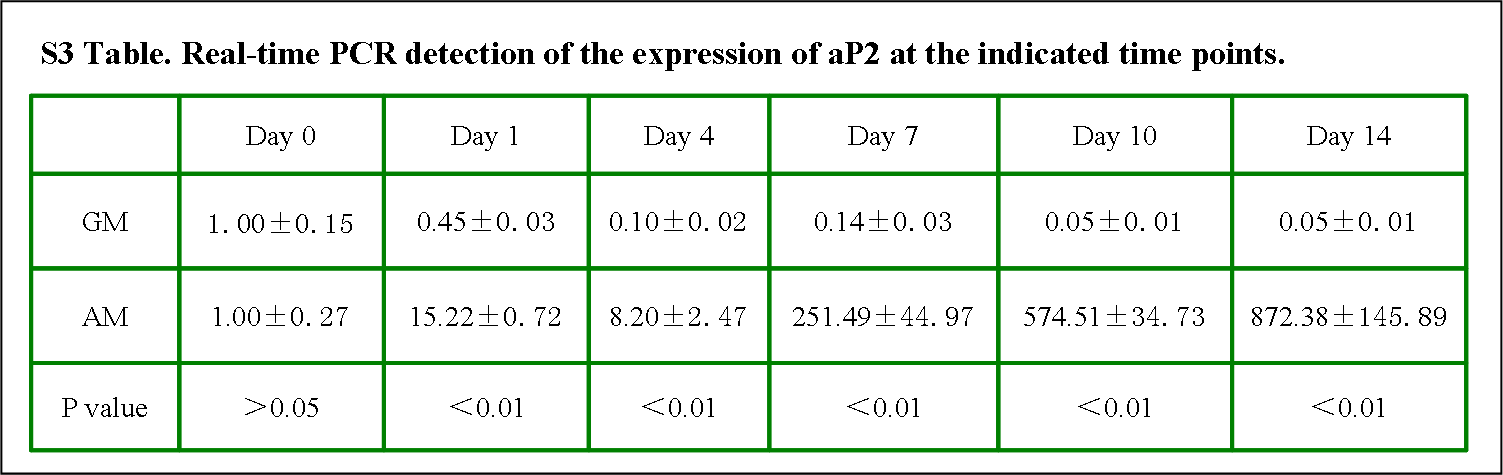

Supplement: S3 Table — (TIF) [file pone.0135611.s003.tif]

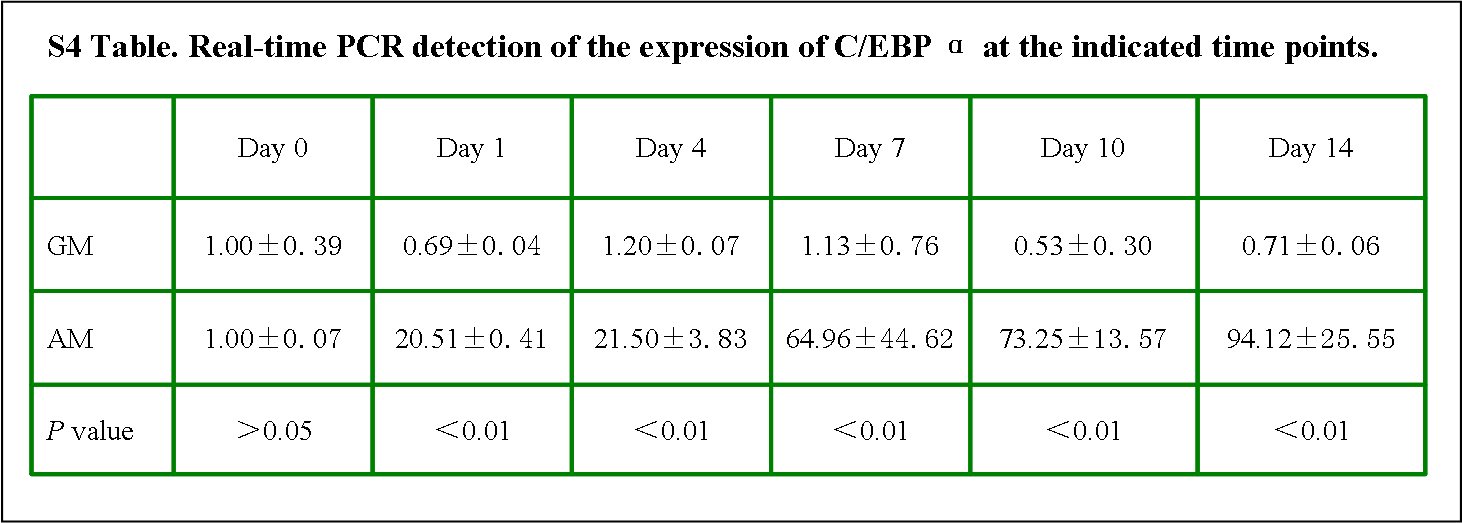

Supplement: S4 Table — (TIF) [file pone.0135611.s004.tif]

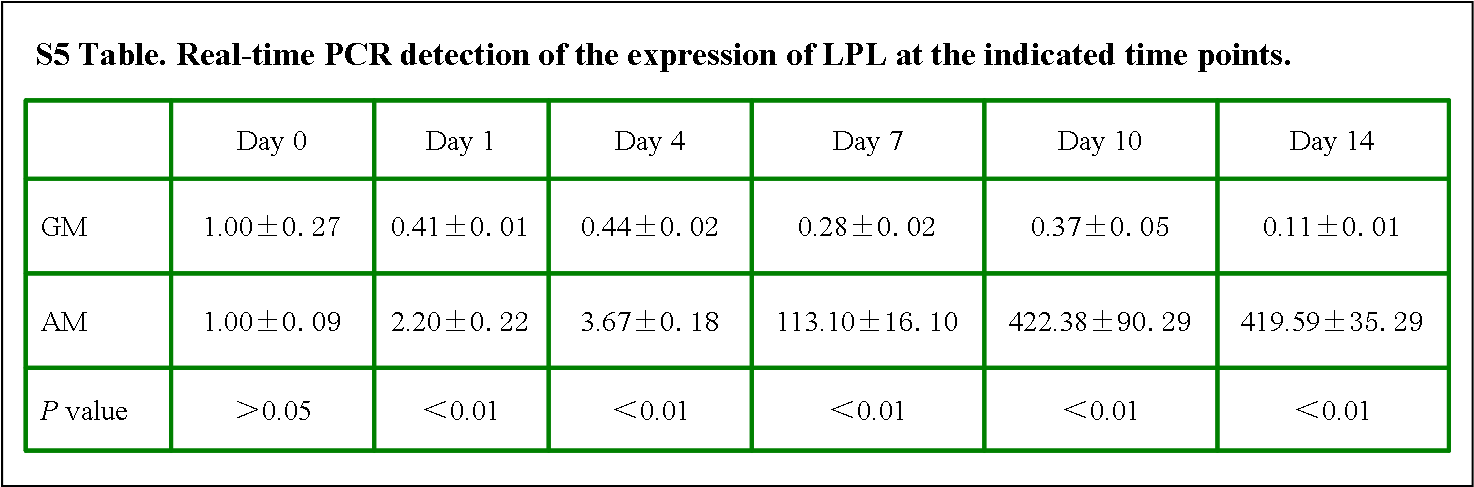

Supplement: S5 Table — (TIF) [file pone.0135611.s005.tif]

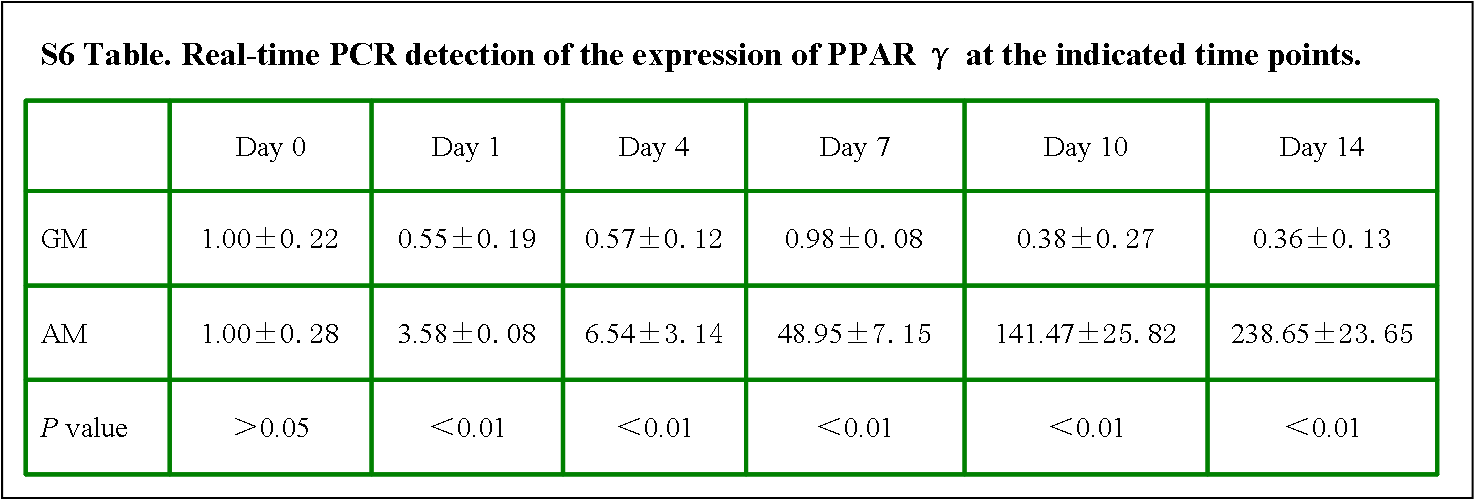

Supplement: S6 Table — (TIF) [file pone.0135611.s006.tif]

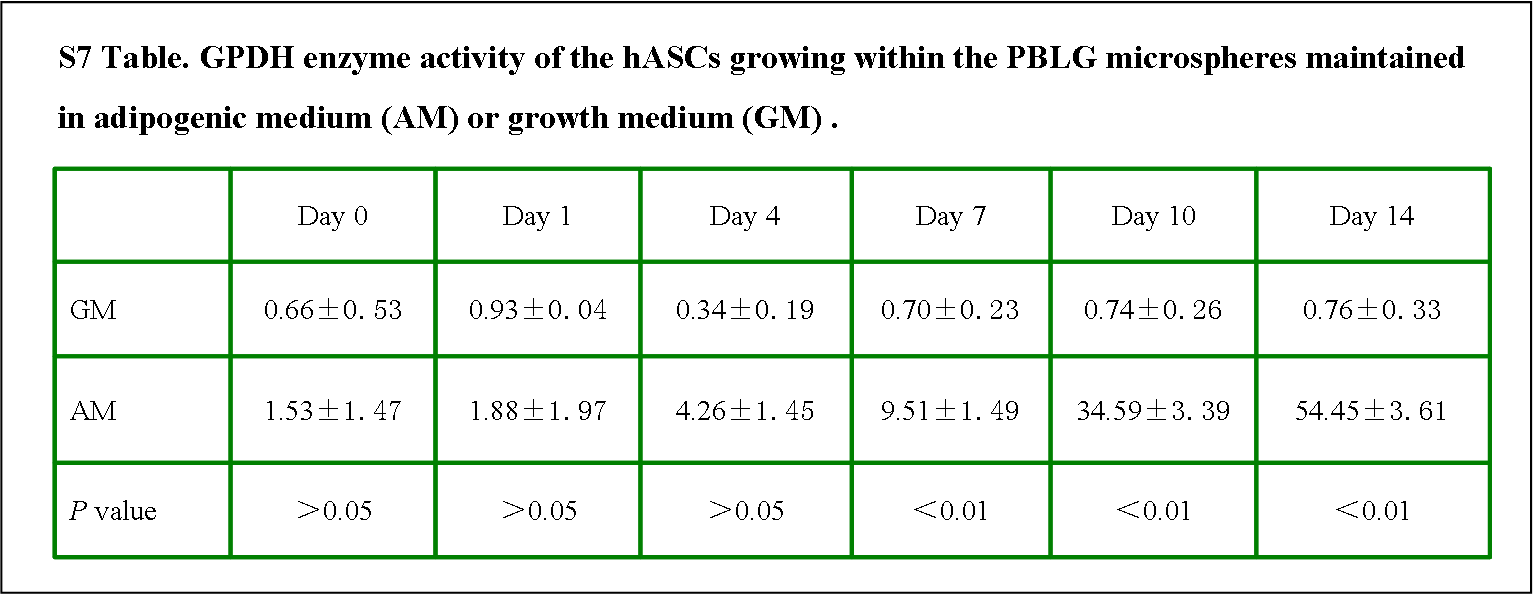

Supplement: S7 Table — (TIF) [file pone.0135611.s007.tif]

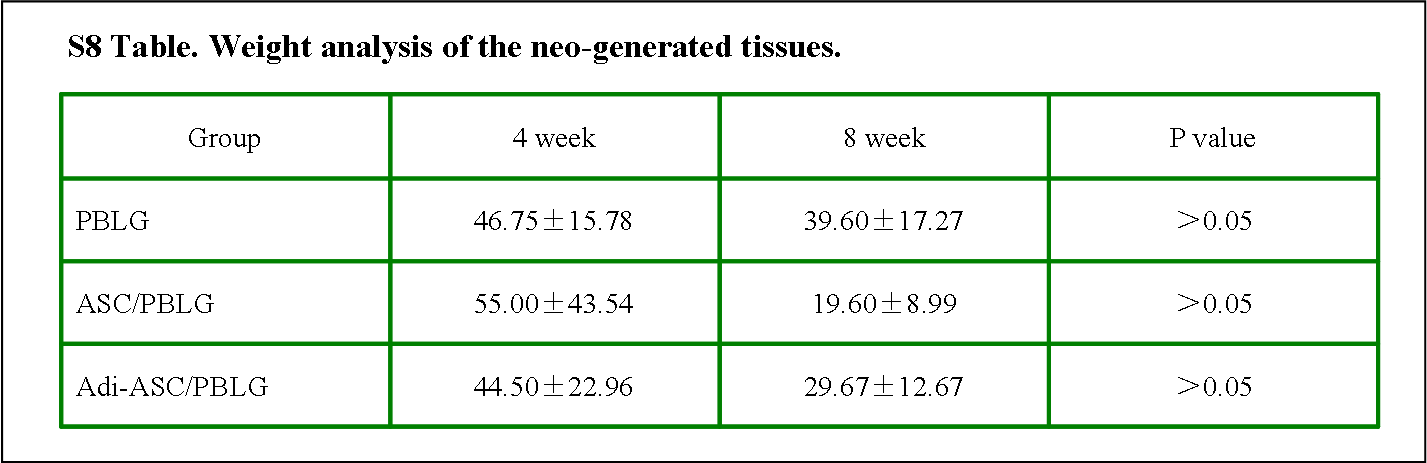

Supplement: S8 Table — (TIF) [file pone.0135611.s008.tif]

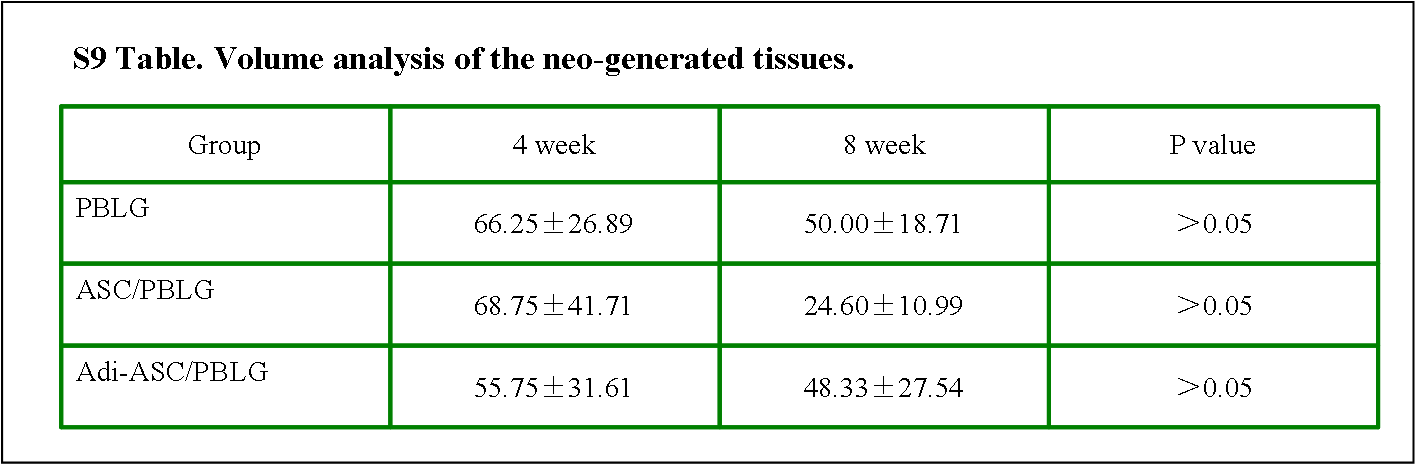

Supplement: S9 Table — (TIF) [file pone.0135611.s009.tif]

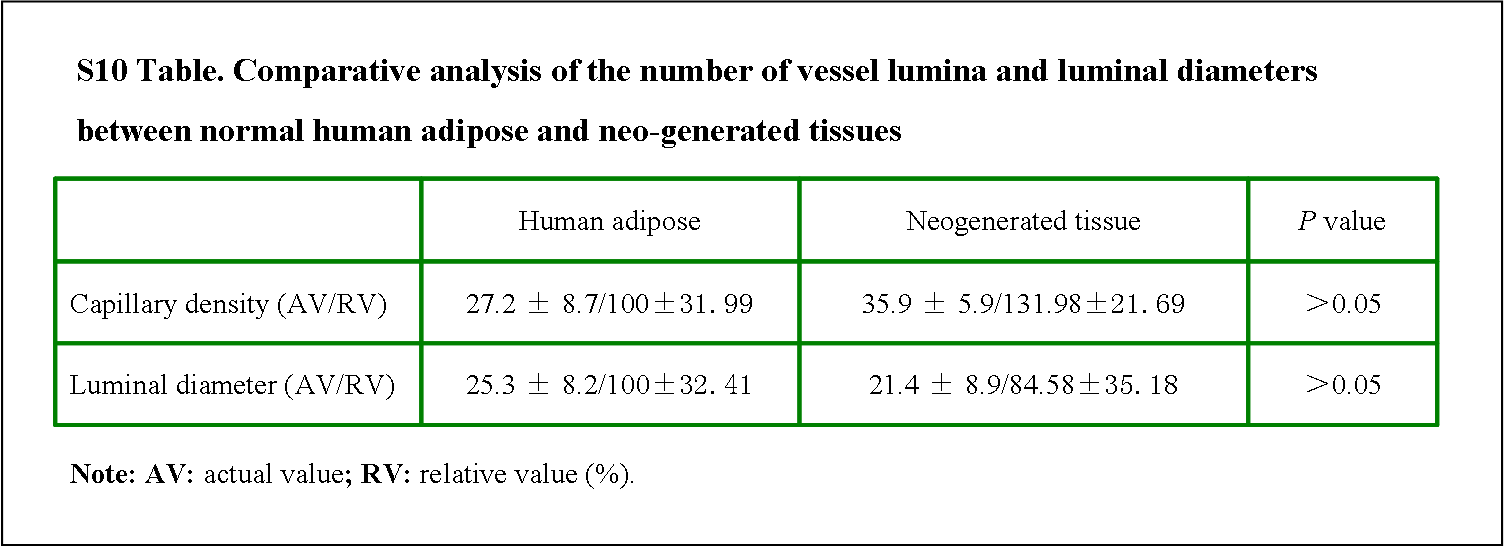

Supplement: S10 Table — (TIF) [file pone.0135611.s010.tif]

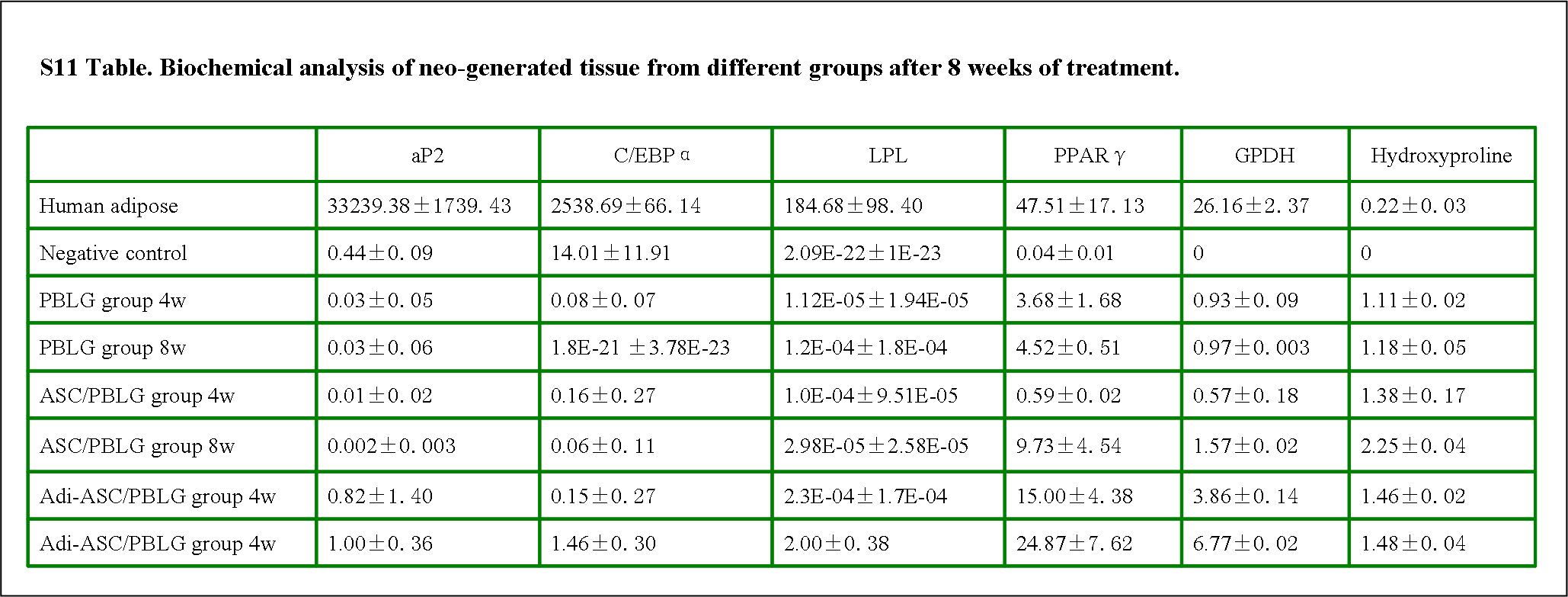

Supplement: S11 Table — (TIF) [file pone.0135611.s011.tif]
